# Identifying and prioritizing lower value services from Dutch specialist guidelines and a comparison with the UK do-not-do list

**DOI:** 10.1186/s12916-016-0747-7

**Published:** 2016-11-25

**Authors:** Joost Johan Godert Wammes, M. Elske van den Akker-van Marle, Eva W. Verkerk, Simone A. van Dulmen, Gert P. Westert, Antoinette D. I. van Asselt, R. B. Kool

**Affiliations:** 1 Radboud University Medical Center, Radboud Institute for Health Sciences, IQ Healthcare, 114 IQ Healthcare, PO Box 9101, Nijmegen, 6500 HB The Netherlands; 2 Department of Medical Decision Making, Leiden University Medical Centre, Albinusdreef 2, Postbus 9600, Leiden, 2300 RC The Netherlands; 3 Department of Epidemiology, University Medical Centre Groningen, Groningen, The Netherlands; 4 Department of Pharmacy, University of Groningen, Antonius Deusinglaan 1, Groningen, 9713 AV The Netherlands

**Keywords:** Low-value, De-adoption, Disinvestment, Waste, Guideline, Choosing Wisely, De-implementation, Medical reversal

## Abstract

**Background:**

The term ‘lower value services’ concerns healthcare that is of little or no value to the patient and consequently should not be provided routinely, or not be provided at all. De-adoption of lower value care may occur through explicit recommendations in clinical guidelines. The present study aimed to generate a comprehensive list of lower value services for the Netherlands that assesses the type of care and associated medical conditions. The list was compared with the NICE do-not-do list (United Kingdom). Finally, the feasibility of prioritizing the list was studied to identify conditions where de-adoption is warranted.

**Methods:**

Dutch clinical guidelines (published from 2010 to 2015) were searched for lower value services. The lower value services identified were categorized by type of care (diagnostics, treatment with and without medication), type of lower value service (not routinely provided or not provided at all), and ICD10 codes (international classification of diseases). The list was prioritized per ICD10 code, based on the number of lower value services per ICD10 code, prevalence, and burden of disease.

**Results:**

A total of 1366 lower value services were found in the 193 Dutch guidelines included in our study. Of the lower value services, 30% covered diagnostics, 29% related to surgical and medical treatment without drugs primarily, and 39% related to drug treatment. The majority (77%) of all lower value services was on care that should not be offered at all, whereas the other 23% recommended on care that should not be offered routinely. ICD10 chapters that included most lower value services were neoplasms and diseases of the nervous system. Dutch guidelines appear to contain more lower value services than UK guidelines. The prioritization processes revealed several conditions, including back pain, chronic obstructive pulmonary disease, and ischemic heart diseases, where lower value services most likely occur and de-adoption is warranted.

**Conclusions:**

In this study, a comprehensive list of lower value services for Dutch hospital care was developed. A feasible method for prioritizing lower value services was established. Identifying and prioritizing lower value services is the first of several necessary steps in reducing them.

**Electronic supplementary material:**

The online version of this article (doi:10.1186/s12916-016-0747-7) contains supplementary material, which is available to authorized users.

## Background

Quality of healthcare is reflected by “*the degree to which health services for individuals and populations increase the likelihood of desired health outcomes and are consistent with current professional knowledge*” [1]. In accordance with this definition, evidence-based medicine means that good medical practices are replaced by better ones when robust scientific evidence becomes available and practices that are outdated or proven invaluable to patients are de-adopted. This ideal world is in sharp contrast with current medical practice [2, 3].

Current practice is not always high-value or evidence based. Lower value or lower quality of care may either be classified into misuse, overuse, or underuse of healthcare services [4]. The focus of this paper is overuse, which occurs when a healthcare service is provided under circumstances in which its potential for harm exceeds the possible benefit [4]. In our study we also include (cost-)ineffective care, inappropriate timing of care, or care not in line with the patients’ wishes as lower value services. Many questions remain about the size of the problem. However, scientific literature suggests that overuse represents between 10% and 30% of provided services, of which a part is lower value care, resulting in worse outcomes including death and unnecessary costs [2, 3, 5]. We consider these services as lower value services, because they have no net value for the patient and de-adoption – a substantial reduction of providing or using the service in daily medical practice – is warranted.

During the last decade, efforts have been undertaken to de-adopt lower value services. UK’s National Institute for Health and Care Excellence (NICE) started working on de-adoption in 2005 [6], resulting in the ‘do-not-do list’ [7]. In the US, the National Physician Alliance started developing ‘Top Five’ lists since 2009 and initiated the Choosing Wisely initiative in 2012 [8]. Australian activities were centered on the Medicare Benefits Schedule [9]. The basis of these programs is usually a (long) list of lower value services and sometimes a prioritization process to identify candidates for de-adoption [9, 10].

The methods for creating these lists are diverse, and prioritization based on impact proves to be difficult. For example, Choosing Wisely lists varied widely in potential impact on daily care and spending, and specialist societies tended to list colleague specialties’ services as lower value [8]. UK research has shown additional challenges, including a lack of reliable evidence on the clinical merits of many services [11]. A prominent problem in overuse is that interventions which are high-value for a given subpopulation are inappropriately applied to other populations [12]. Candidate lists tend to be large and the potential gains in health and cost vary widely across lower value services. Therefore, as resources for de-adoption are limited, prioritization of lower value services for de-adoption is warranted.

To conclude, there is need for an objective approach to identify and prioritize lower value services for practical de-adoption [11]. This article describes the development of a list of lower value services identified from 193 Dutch clinical practice guidelines, published between 2010 and 2015. The list was developed with the aim to provide a comprehensive list of lower value services for Dutch hospital care. Furthermore, our list was compared with the NICE do-not-do list on several aspects, including types of care and patient groups. Finally, the feasibility of prioritizing the list was studied. We hypothesized the prevalence of a disease and disease burden (a rationale for choice of criteria is given in the discussion) could serve as robust criteria for prioritization.

## Methods

### Development of lower value services list

Dutch guidelines contain specific recommendations to ensure that lower value care is not offered, or only applied to specific subpopulations or under limiting conditions. In the current study we identified these do-not-do recommendations. We have limited the analysis to the most recent and up to date guidelines published between January 2010 and May 2015 by the scientific societies, as Dutch guidelines are recommended to be revised every 5 years [13]. The guidelines were taken from a guideline database hosted by the Dutch Association of Medical Specialists (www.kwaliteitskoepel.nl) covering (mental) hospital care.

Firstly, we randomly selected 11 guidelines which were fully read by four researchers (SD, EV, JW and MEAM) to identify recommendations on care that should not be offered and care that should not be offered routinely. For each do-not-do recommendation identified, we listed whether the key term identifying the do-not-do recommendation was one of the search terms applied by NICE in the ‘do-not-do’ study (for example, ‘discontinued’, ‘should not’, ‘do not’ [14]) or a new term that should be added (e.g., ‘omit’). Recommendations that focused on too little use of care (underuse) were not included. For example: “Restraint is not necessary when starting opioids and will lead to a substantial deterioration in quality of life by the experienced severe shortness of breath” (Guideline: Palliative care for people with chronic obstructive pulmonary disease). Finally, recommendations that focus on organization of care were not included. For example, “It is not recommended that professionals who have no experience with patients/offenders with antisocial personality (disorder) address the issue of the committed violence” (Guideline: Domestic violence in children and adults). A fifth researcher (RBK) was consulted in case of no consensus.

Furthermore, the specific section of the guideline in which the do-not-do recommendation was written was identified. The standard format of guidelines contains five sections: clinical question, recommendations, substantiation, considerations, and justification. As in the first five guidelines, all the recommendations were found in the sections ‘recommendations’ and ‘considerations’ of the guidelines; subsequently, only these sections of the electronic/PDF copy of a guideline were searched with the terms from Table 1.

**Table 1:** Shortlist search terms

Dutch [English translation]	English
Niet [Not]	Discontinue/discontinuation
Geen [No]	Not
Stop [Stop]	No
Onvoldoende [Insufficient]	Ineffective
Zelden [Seldom]	Uncertain
Alleen [Only]	Avoid
Kosten [Cost]	Rarely
Vermijd/Vermeden [Avoid]	Stop
Achterwege [Omit]	
Onnodig [Unnecessary]	
Afgeraden [Discourage]	
Ontraden [Dissuade]	
Staken/Gestaakt [Cease]	

Another nine guidelines were independently screened by the four researches (SD, MEAM, EV and JW) to determine the inter-rater reliability. Inter-rater reliability was analyzed by calculating Fleiss’ Kappa (k) for multiple raters [15].

Using this method, the other guidelines were screened (in total 193), and any ambiguities were discussed with another researcher until consensus was reached. When guidelines were not constructed according to the standard format and therefore did not contain the paragraphs with recommendations and considerations, they were fully screened. For each do-not-do recommendation identified we assessed whether the care should not be offered at all or should not be offered routinely to all patients and what type of care the recommendation was about: diagnosis, treatment without medication, treatment with medication, and a residual category.

Guidelines that have been published in English were screened with English terms. Patient versions of guidelines were not included and also addenda to guidelines with original publication date before 2010 were excluded.

### Connection with International Classification of Disease, Tenth Edition (ICD10) code

The lower value services described in the do-not-do recommendations were provided with an ICD10 code by searching within the ICD10 encoding [16] on the condition in question. When necessary, additional information was sought in the guideline from which the lower value service originated and/or Wikipedia. If the lower value service was related to two (or more) conditions, the guideline topic was selected for the ICD10 coding. For example, the guidance “European Guidelines on cardiovascular disease prevention in clinical practice” included the recommendation “Beta-blockers and thiazide diuretics are not recommended in hypertensive patients with multiple metabolic risk factors increasing the risk of new-onset diabetes”. This recommendation was categorized to the ICD10 code for hypertensive diseases. If the patient population receiving the lower value service could not be related to an ICD10 code, for example, in the case of prevention in a healthy population, then the ICD10 code of the disease prevented was chosen. For example, the lower value service “Do not use throat swabs when investigating for possible meningococcal disease” concerns the population with suspected meningococcal disease. Since there is no ICD10 code for this population, the ICD10 code of meningococcal disease was chosen. Complex cases were discussed between two researchers until consensus was reached. ICD10 codes were then aggregated to ICD10 chapters, the highest level of categorization in ICD10.

### Comparison with NICE do-not-do database

In the development of NICE guidelines, clinical practices were identified which should not be used at all or should not be used routinely. These practices have been collected in the do-not-do database [7]. NICE made an Excel file of the database (dated September 29, 2015) available to us upon request. We compared the average number of do-not-do recommendations per NICE guideline with the Dutch number. Furthermore, for each recommendation from the NICE do-not-do database we assessed whether the care should not be offered at all or should not be offered routinely and what type of care was concerned (diagnosis, treatment without medication, treatment with medication). Finally, the same procedure with respect to assigning ICD10 codes was followed.

### Prioritization

Prioritization of conditions for further research on lower value services for de-adoption was done by aggregating the lower value services described in the do-not-do recommendations by ICD10 codes, as the data for prioritization were only available at this level of aggregation and not for individual lower value services. Per ICD10 code we identified prevalence estimates and disease burden as available in the Global Burden of Disease studies [17] (a detailed description of the methodology is given in Additional file 1: Appendix 1). Prioritization was based on the number of lower value services per ICD10 code, prevalence and burden of disease (expressed in Years Lived with Disabilities (YLD) and Disability Adjusted Life-Years (DALY)). Each criterion was categorized in four groups according to level. Per criterion, the group with the highest levels was assigned four points. Subsequently, the ICD10 codes were prioritized by the sum of scores for the number of lower value services, prevalence, YLD, and DALY (Method 1), with the highest score (up to 16) indicating the highest priority for de-adoption. As we were interested in the impact of burden of disease measures on prioritization (both YLD and DALY reflect burden of disease) we omitted these criteria in sensitivity analyses, and the prioritization was repeated for the sum of the number of lower value services and prevalence (Method 2; maximum score 8). For the NICE do-not-do database the same prioritization was performed, using UK-specific data on prevalence, YLD and DALY. In Additional file 1: Appendix 1, a full description of the prioritization methodology is given.

## Results

### Descriptive Dutch list of lower value services

In total, 1366 lower value services were extracted from the 193 Dutch guidelines on (mental) hospital care, implying that each guideline contained, on average, 7.1 (modus = 0; median = 5; maximum = 45) lower value services. Of these guidelines, 29 did not contain any lower value services. The inter-rater reliability was 0.803 (Fleiss k), indicating a substantial agreement [18]. Table 2 shows the average number of lower value services per guideline between 2010 and 2015. The number of guidelines published in 2014 and 2015 was relatively low because of the ending of a subsidy program. The majority of lower value services was, if necessary after deliberation within the project group, successfully linked to an ICD10 code. In 98 cases (<8%), no ICD10 code could be assigned, predominantly because the recommendation was ambiguous concerning the patient group, or the patient group was insufficiently specific (e.g., ‘essentially, laparoscopic surgery does not require different fluid management than open surgery’).

**Table 2:** Number of lower value services per year in Dutch guidelines

Year	Number of guidelines published	Number of lower value services	Average number of lower value services per guideline
2010	61	357	5.85
2011	41	249	6.07
2012	44	347	7.89
2013	35	312	8.91
2014	2	45	22.5
2015	6	59	9.83

Of the lower value services, 415 (30%) related to diagnostics, such as ‘There is no place for FDG-PET in the detection of micro metastases’ (guideline anus carcinoma, Dutch list); 399 lower value services (29%) related to non-drug treatment, such as ‘The insertion of a pulmonary artery catheter (PAC) in case of acute heart failure is rarely needed’ (guideline heart failure, both in Dutch list and NICE database). Finally, 527 lower value services (39%) related to drug treatment, such as ‘Methotrexate is not recommended for hidradenitis suppurativa’ (guideline acneiform dermatoses, Dutch list). The remaining 25 (2%) lower value services did not fit into these categories (e.g., vaccination or recommendations on referral and discharge procedures). The majority (77%) of all lower value services concerned care that should not be offered at all, whereas the other 23% recommended on care that should not be offered routinely.

**Fig. 1:**
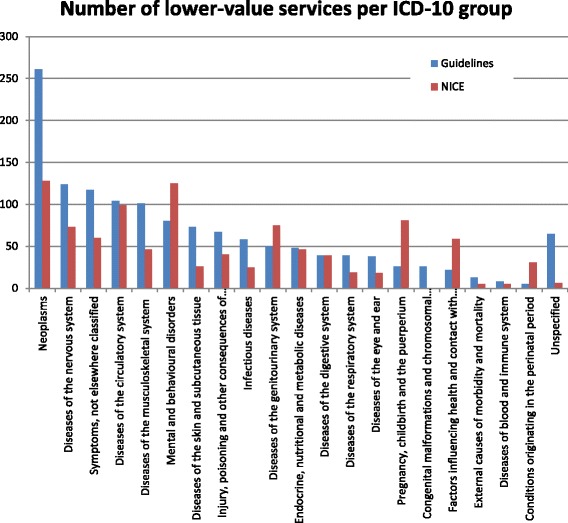
Number of lower-value services per ICD10 group for Dutch guidelines and NICE do-not-do list

Figure 1 shows the number of lower value services identified per ICD10 chapter. For the Dutch guidelines, ‘neoplasms’ and ‘diseases of the nervous system’ are the most frequent chapters, followed by ‘symptoms, signs and abnormal clinical and laboratory findings – not elsewhere classified’, ‘diseases of the circulatory system’, ‘diseases of the musculoskeletal system and connective tissue’, and ‘mental and behavioral disorders’. Relatively few lower value services were found in ICD10 chapters ‘external causes of morbidity and mortality’, ‘conditions originating in the perinatal period’, and ‘diseases of the blood and blood-forming organs and certain disorders involving the immune mechanism’.

### Comparison with NICE do-not-do recommendations

The database contained 188 guidelines in which 1006 do-not-do recommendations (lower value services) were found. The UK guidelines thus covered relatively few lower value services, on average, 5.4 (modus = 1; median = 3; maximum = 32) per guideline. UK guidelines covered slightly fewer lower value services related to diagnostics (28%) and non-drug treatment (25%), and relatively many lower value services related to drug treatment (46%). In addition, UK lower value services less likely described care that should not be offered at all (68%), whereas the other 32% recommended care that should not be offered routinely. Finally, UK do-not-do recommendations more frequently covered mental and behavioral disorders, diseases of the genitourinary system, pregnancy, childbirth, and the puerperium (Fig. 1).

### Prioritization of Dutch lower value services

**Fig. 2:**
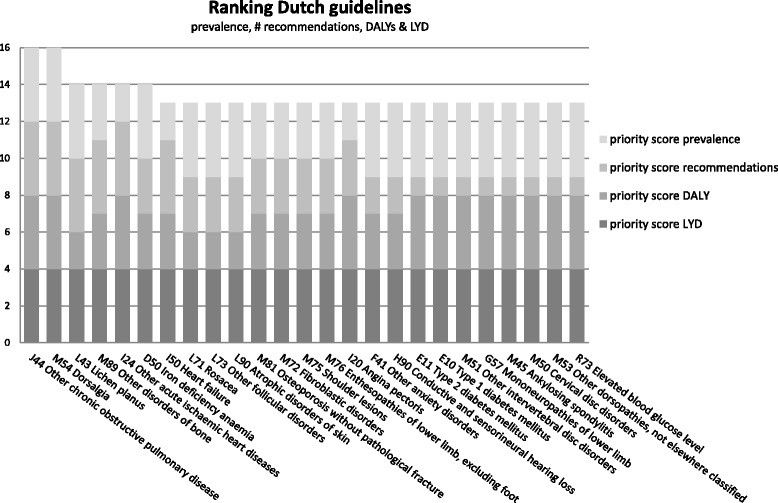
Ranking results from Dutch guidelines (method 1)

**Fig. 3:**
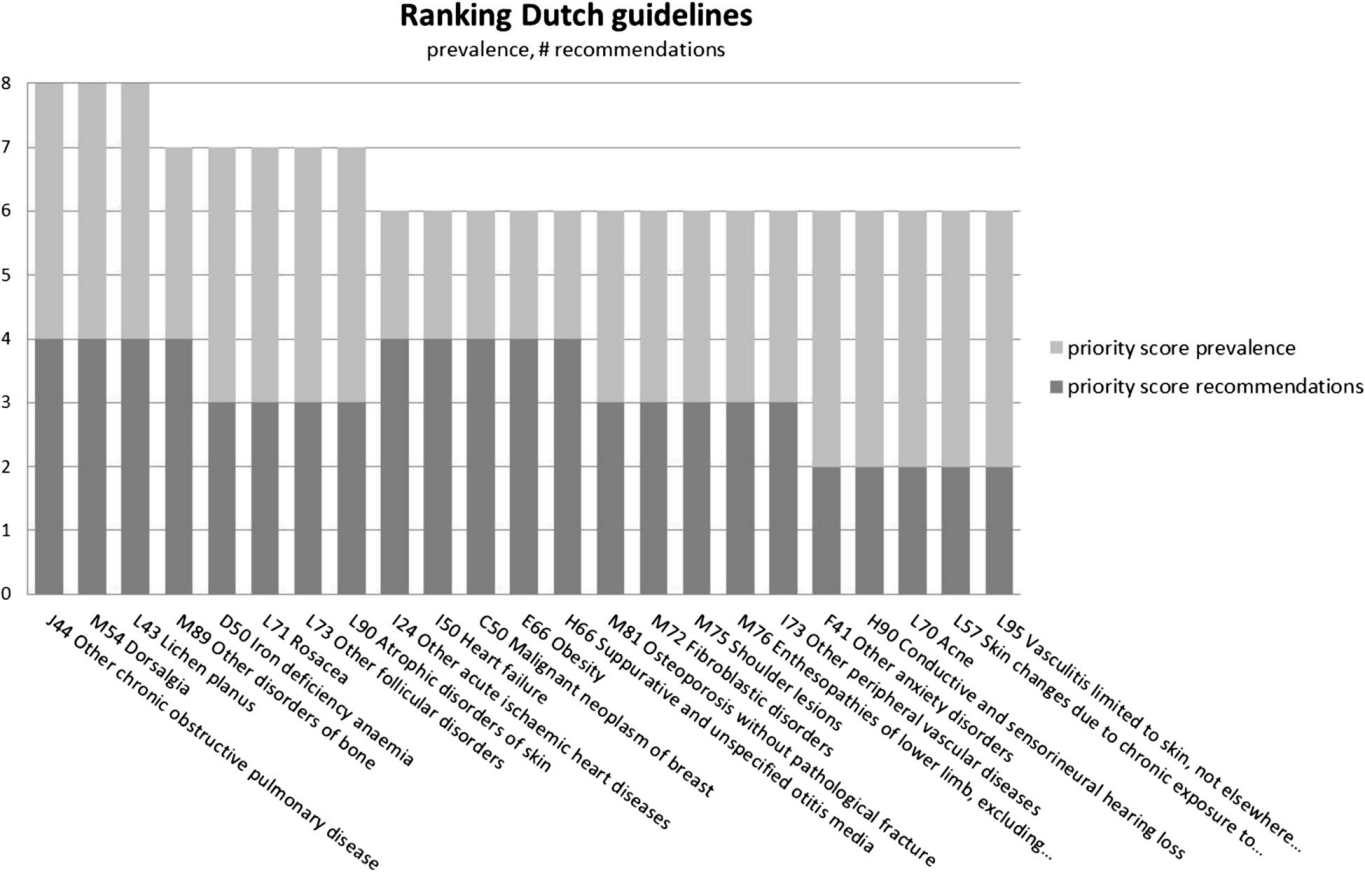
Ranking results from Dutch guidelines (method 2)

As mentioned, the ranking was performed according to two different strategies. The results of the ranking by prevalence, DALY, YLD and number of recommendations (method 1) is represented in Fig. 2. Both dorsalgia (back pain) and other chronic obstructive pulmonary diseases were assigned the maximum score of 16, followed by other acute ischemic heart diseases, iron deficiency anemia, lichen planus, and other disorders of bone (in particular the complex regional pain syndrome type 1), each of which scored 14 points. Furthermore, out of the top-25 prioritized ICD10 codes, 10 (40%) are in chapter M, i.e., diseases of the musculoskeletal system and connective tissue. When the ranking was performed by only prevalence and number of recommendations (method 2, Fig. 3), three diseases obtained the maximum score, i.e., dorsalgia, other chronic obstructive pulmonary disease, and lichen planus.

Generally speaking, neoplasm ICD10 codes receive a more modest priority when number of recommendations and prevalence are the only criteria for prioritization, but receive higher priority when burden of disease criteria are included. Ranking results for UK lower value services are provided in Additional file 1: Appendix 2.

## Discussion

In this study, we developed a comprehensive list of lower value services for Dutch hospital care and studied the feasibility of prioritizing the list. In addition, we repeated the descriptive analyses and prioritization for the UK do-not-do database. In total, 1366 lower value services were extracted from 193 Dutch guidelines. Of the lower value services 30% covered diagnostics, 29% related to non-drug treatment, and 39% to drug treatment. The majority (77%) of all lower value services was on care that should not be offered at all, whereas the other 23% recommended on care that should not be offered routinely. ICD10 chapters that included most lower value services were neoplasms and diseases of the nervous system. Further research and policy aimed at reducing lower value services are highly warranted. A recent Dutch study showed avoidable costs are evident in healthcare: about 60 million euro can be saved in the Netherlands, when 23 lower value surgical procedures – actual use approximately 11,800 in the Netherlands – are no longer performed [19].

The prioritization processes revealed several ICD10 codes with relatively high prevalence and disease burden where lower value services most likely occur and de-adoption is warranted, including back pain, chronic obstructive pulmonary diseases, acute ischemic heart diseases, iron deficiency anemia, lichen planus, disorders of bone, and malignant neoplasms of bronchus and lung. These findings are relevant, given the corresponding opportunities for further research. However, this prioritization should be interpreted with caution, it does not prove lower value services are actually provided to these groups. Rather, based on robust criteria, we recommend further research into the presence of lower-value services in these conditions.

The Dutch and UK list show similarities as well as differences. Dutch guidelines appear to contain more lower value services than the UK guidelines (7.1 on average vs. 5.4, respectively). These data suggest Dutch guideline developers might be more aware of the existence of lower value services or might consider incorporating do-not-do recommendations in guidelines more important than their UK colleagues. However, differences in followed methodology might have spurred this difference. We only included guidelines published between 2010 and 2015, whereas NICE started in 2005, and we have shown an increase in number of do-not-do recommendations per year. Moreover, we also included recommendations from consideration sections. This probably makes the Dutch list more comprehensive.

The development of a comprehensive list of lower value services and prioritization is only the first of several necessary steps in actually reducing lower value services, starting with measuring the actual use of lower value services. As discussed above, many uncertainties remain about the prevalence of lower value services. Estimates for the Netherlands date back to the ‘90s [3], or have to be gauged from case studies. Like Morgan et al. [5], we support routine monitoring of potential “outbreaks” in use of diagnostics and treatment methods and variation in routine care. Such an approach entails large scale measurements using real time administrative data with sufficient clinical detail to assess appropriateness of care and risk adjustment, which are not yet available in the Netherlands. De Vries et al. [20] recently identified 115 lower value care measures, which mainly focused on the cure sector. Apart from these indicators, our database could be used for developing new and valid indicators for lower value care.

Early evidence shows that dissemination of recommendations alone is not sufficient to ensure de-adoption, and that additional specific interventions are required. For example, a first evaluation of the Choosing Wisely initiative showed marginal reductions of use, if any [21], whereas Schwartz et al. [22] showed that alternative payment models with global budgets successfully discouraged overuse. Several papers discuss interventions or provide roadmaps for reducing overuse or promoting/advancing de-adoption [5, 23, 24]. Most notably, Niven et al. [24] proposed a conceptual model for the process of de-adoption; which shares much of the original Knowledge-to-Action Cycle [25]. The proposed framework emphasizes in-depth analyses of barriers and facilitators, which is deeply grounded in adjacent fields such as implementation science [26]. Paprica et al. [27] underlined that stakeholders should be involved in de-adoption. In their analysis, they point to the trinity by Lomas et al. [28] – medical effectiveness research (context-free scientific evidence), social science-oriented research (context-sensitive scientific evidence), and the expertise, views, values, and realities of stakeholders (colloquial evidence) – and show that colloquial evidence has a major influence in de-adoption. Local stakeholder involvement is therefore pivotal in de-adoption initiatives. In this study, we focused on identifying and prioritizing lower value services. This process is central to the Niven framework and is ideally performed concomitant with stakeholder engagement. Stakeholders could, for example, participate in choosing and weighting prioritization criteria. In addition, expert panels could be employed to further rank our list of lower value services on appropriateness of the services and priority for de-adoption [29].

In the Netherlands, the exact above formula for reducing lower value care is being followed. The Dutch Federation of University Medical Centers recently initiated a 4-year program for reducing lower value services. The current study is the first outcome of this project and, in June 2016, all eight university hospitals commenced local de-adoption pilot projects. The current list and prioritization contributed to selecting appropriate conditions and lower value services for de-adoption. The list will be integrated with the guideline database (www.richtlijnendatabase.nl) of the Dutch Association of Medical Specialists. On this website, all lower value service recommendations will be highlighted, and special attention will be paid to the fact that, in these cases, not acting is a better solution.

### Limitations

The methodology we developed for this study has a number of limitations, for a large part related to ambiguity in guideline recommendations and lacking data. Ambiguity in guideline recommendations sometimes made it difficult to discern lower value services, or to distinguish between care that should not be offered at all, and care that should not be offered routinely. In some cases, it was explicitly mentioned that care was not recommended, whereas, in others, this was less explicit. For example, “No recommendations can be given for the use of tramadol or oxycodone in the emergency medical treatment on the basis of the emergency care literature” (Guideline: Pain management in emergency care chain). These recommendations have been included as the context shows that application is not indicated. To cope with ambiguous recommendations, regular meetings were held to discuss disputable items until consensus was reached. Nevertheless, ambiguity of guideline recommendations or ambiguous populations may have biased our findings.

The Dutch list of lower value services was developed to comprehensively cover lower value services in Dutch hospital care. We restricted inclusion of guidelines to the period from 2010 until May 2015, as Dutch guidelines are recommended to be revised every 5 years [13]. As a result, we could not take into account important conditions or diseases covered by older guidelines, by guidelines published after May 2015 or not covered by guidelines at all. Furthermore, we might have missed some lower value services that lacked one of the keywords we identified. We therefore recommend to routinely update the list and to update the list of keywords.

Ideally, lower value services are prioritized based on the following criteria: the availability of evidence that a service is ineffective or harmful, patient safety, potential health and cost impact of de-adoption, availability of alternative practices [30], and the actual use of the lower value service. Clarifying such information for over 1000 lower value services proved impossible and much of such detailed information is currently lacking. We therefore developed alternative criteria as close as possible to the criteria proposed by Elshaug et al. [30]. Notwithstanding the methodological hurdles and data problems, we consider the prioritization results robust for singling out new and valid information besides the list itself, and both are useful for informing de-adoption programs. Finally, in this study, stakeholders were not involved, which should be a next step in the process of de-adoption. The prioritization results may be important input for this consultation step.

## Conclusions

In this study, a comprehensive list of lower value services for Dutch hospital care was developed. The majority of lower value services covered care that should not be offered at all; 30% of lower value services covered diagnostics, 29% were related to non-drug treatment, and 39% to drug treatment. Comparing the list with its UK counterpart revealed that Dutch guidelines appear to contain more lower value services than the UK guidelines. Finally, a feasible method for prioritizing lower value services was established. The development of a comprehensive list of lower value services and prioritization is only the first of several necessary steps in reducing lower value services.

## Additional file

Additional file 1: Prioritization methodology and UK results are presented in appendix 1 and 2. (DOCX 230 kb)

## Abbreviations

DALY: disability-adjusted life year
ICD: International Classification of Disease
NICE: National Institute for Health and Care Excellence
UK: United Kingdom
YLD: years lost due to disability
