# Supplementary material for: Identifying and prioritizing lower value services from Dutch specialist guidelines and a comparison with the UK do-not-do list
Source: BMC Med. 2016 Nov 25;14:196. doi: 10.1186/s12916-016-0747-7 (PMC5123317; doi:10.1186/s12916-016-0747-7)
Supplement: Additional file 1: — Prioritization methodology and UK results are presented in appendix 1 and 2. (DOCX 230 kb) [file 12916_2016_747_MOESM1_ESM.docx]

**Additional file**

**Appendix 1. prioritization methodology**

4a. Connection with Global Burden of Disease

Since information on prevalence and disease burden by ICD10-code is not systematically available in the Netherlands, it was decided to use the Global Burden of Disease (GBD) classification. The GBD is a global classification of disease categories including prevalence, disability adjusted life years (DALYs), years of life lost (YLL) and years lost due to disability (YLD) for each disease category, and is regularly renewed, most recently in 2015^[[1]](#footnote-1)^ with 2013 data. Many of the GBD parameters are available online and by country. Also, information was available which ICD10-codes are covered by each GBD-category. A GBD-category often covers a range of ICD10-codes. For example, the GBD-category "low back pain" consists of a number of ICD10-codes from Chapter VI - Diseases of the nervous system (including disorders of lumbosacral plexus), but also from Chapter XIII - Illnesses of the musculoskeletal system and connective tissue (including instability of spine).

The ICD10-codes were assigned to GBD-categories. If an ICD10-code fitted in more than one GBD category, the category covering the smallest range of ICD10-codes was chosen. Part of the ICD10-codes only fitted in the 'garbage code' category, which is a very broad group. Therefore, for those ICD-10 codes that were assigned to the rest category ‘garbage code’, an alternative GBD-category was sought, if possible. If no ICD10-code was assigned to a lower value service but an ICD10-group or specialism was known, this information was used to find an appropriate GBD-category for the lower value service. Four researchers (MEAM, EV, JW, TA) each took a randomly selected part of the list to assign GBD-categories to ICD10-codes. Equivocal cases were discussed with another researcher until consensus was reached. Not for each GBD-category prevalence figures were known, and to a lesser extent, DALYs and YLDs were missing.

4b. Prioritization of ICD10-codes

To apply a prioritization in the extensive list of lower value services, the following criteria were defined per ICD code: number of lower value services, prevalence, LYD and DALYs. For each criterion four groups were made that were given a priority score. The classification into four groups aimed at having a comparable number of ICD10-codes in each group, as well as obtaining rounded categories (i.e. 500-1000 instead of 439-768). This resulted in the following classification:

Number of lower value services per ICD10-code: >10 (4 points), 5-10 (3 points), 2-5 (2 points), 1 (1 point);

Prevalence (Netherlands 2013;*1000): >1000 (4 points), 500-1000 (3 points), 100-500 (2 points) , <= 100 (1 point);

YLD (Netherlands 2013;*1000): >10 (4 points), 5-10 (3 points), 1-5 (2 points), <=1 (1 point);

DALY (Netherlands 2013;*1000): >100 (4 points), 50-100 (3 points), 10-50 (2 points), <=10 (1 point).

Subsequently, the ICD10-codes were prioritized by the sum of the number of lower value services, prevalence, LYD and DALY (Method 1), the highest score (up to 16) indicating the highest priority. As YLD is part of the DALY, this provides a high priority for ICD10-codes with a high number of YLD. Therefore, the prioritization was repeated for the sum of the number of lower value services and DALYs (Method 2; maximum score 8).

**Appendix 2: UK prioritization results.**


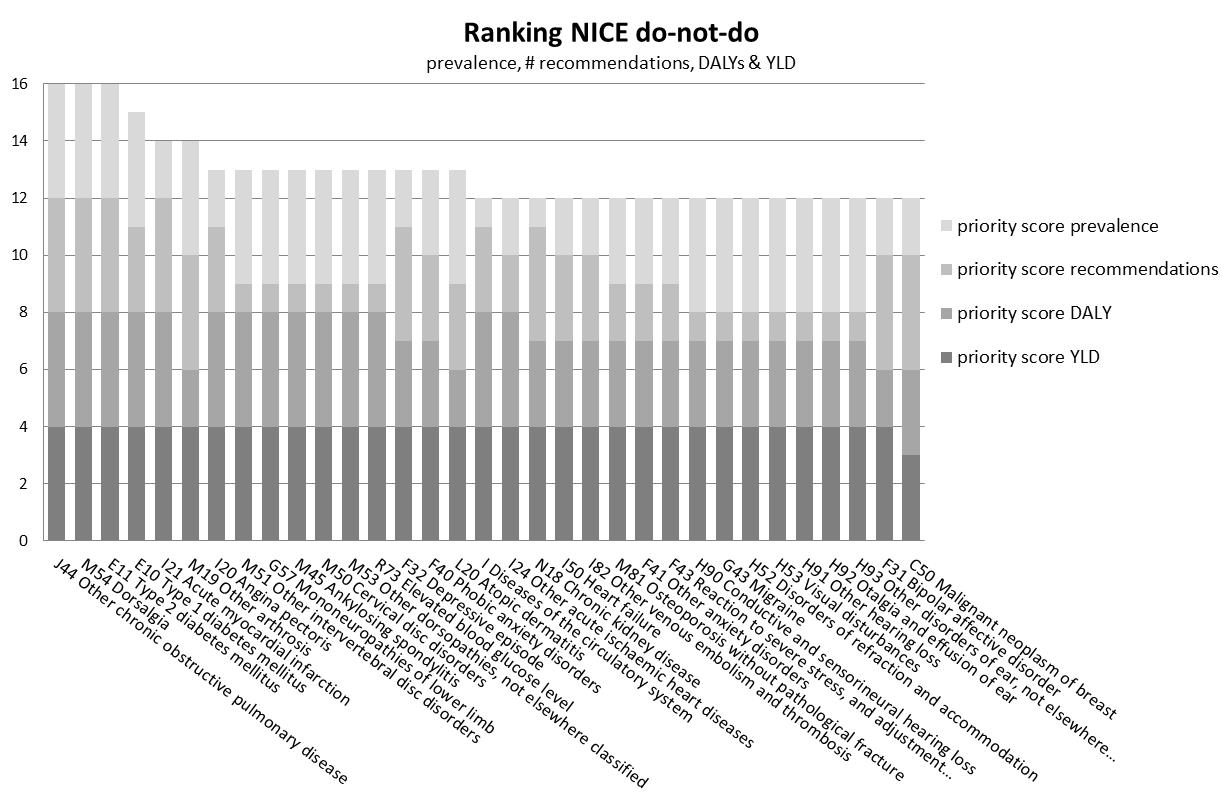


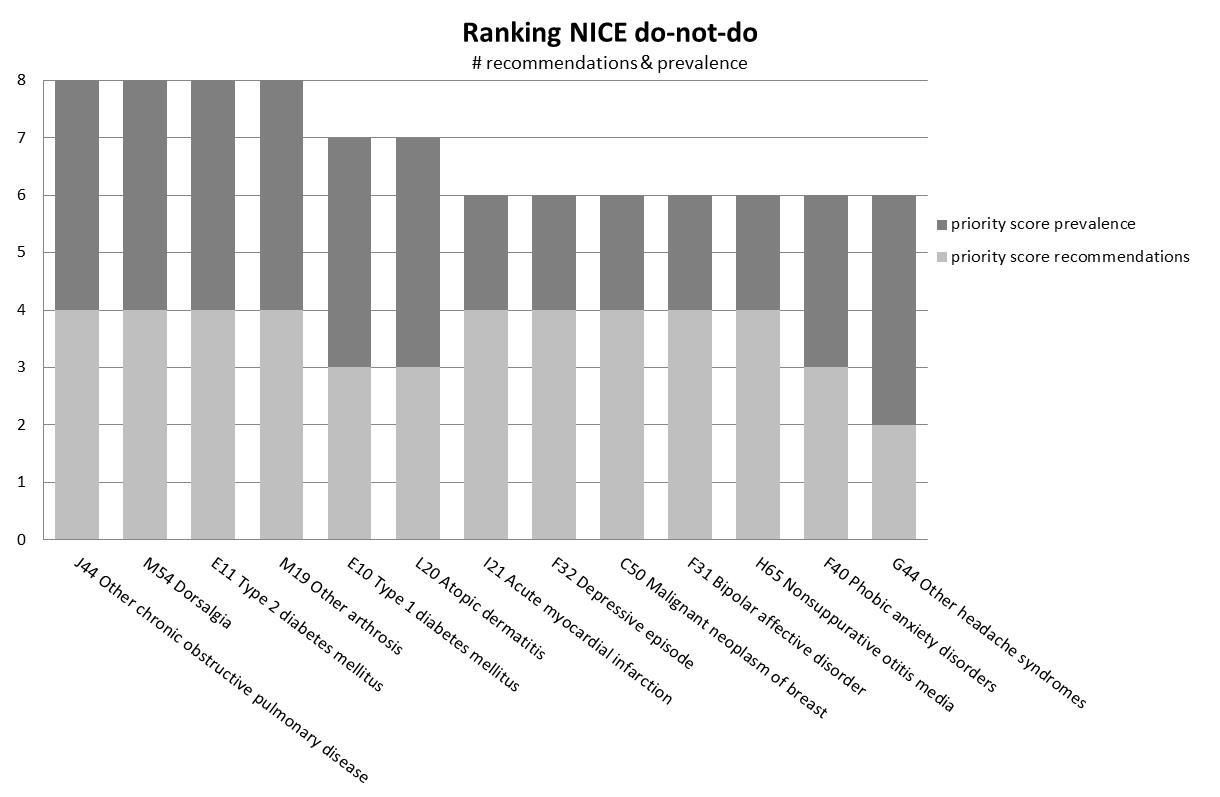


1. Global Burden of disease Study Collaborators 2013; <http://ghdx.healthdata.org/global-burden-disease-study-2013-gbd-2013-data-downloads> Global Burden of Disease Study 2013. Global Burden of Disease Study 2013 (GBD 2013) Incidence, Prevalence, and Years Lived with Disability 1990-2013. Seattle, United States: Institute for Health Metrics and Evaluation (IHME), 2015. [↑](#footnote-ref-1)
